# Exploring the role of endogenous retroviruses in seasonal reproductive cycles: a case study of the ERV-V envelope gene in mink

**DOI:** 10.3389/fcimb.2024.1404431

**Published:** 2024-06-28

**Authors:** Yufei Zhang, Gaofeng Wang, Yanzhu Zhu, Xiaodong Cao, Fang Liu, Huiping Li, Shuying Liu

**Affiliations:** ^1^ College of Veterinary Medicine, Inner Mongolia Agricultural University, Hohhot, China; ^2^ Ulanqab Center for Animal Disease Control and Prevention, Ulanqab, China; ^3^ Institute of Special Animal and Plant Sciences, Chinese Academy of Agricultural Sciences, Changchun, China; ^4^ School of pharmacy, Inner Mongolia Medical University, Hohhot, China; ^5^ College of Veterinary Medicine, Key Laboratory of Basic Veterinary Medicine, Inner Mongolia Agricultural University, Hohhot, China; ^6^ Key Laboratory of Clinical Diagnosis and Treatment Technology in Animal Disease, Ministry of Agriculture, Hohhot, China

**Keywords:** endogenous retroviruses, retrovirus, envelope, mink, seasonal breeder, testis, testis regression

## Abstract

**Introduction:**

Endogenous retroviruses (ERVs), which originated from exogenous retroviral infections of germline cells millions of years ago and were inherited by subsequent generations as per Mendelian inheritance patterns, predominantly comprise non-protein-coding sequences due to the accumulation of mutations, insertions, deletions, and truncations. Nevertheless, recent studies have revealed that ERVs play a crucial role in diverse biological processes by encoding various proteins.

**Methods:**

In this study, we successfully identified an ERV envelope *(env*) gene in a mink species. A phylogenetic tree of mink ERV-V *env* and reference sequences was constructed using Bayesian methods and maximum-likelihood inference.

**Results:**

Phylogenetic analyses indicated a significant degree of sequence conservation and positive selection within the *env*-surface open reading frame. Additionally, qRT-PCR revealed diverse patterns of mink ERV-V *env* expression in various tissues. The expression of mink ERV-V *env* gene in testicular tissue strongly correlated with the seasonal reproductive cycles of minks.

**Discussion:**

Our study suggests that the ERV-V *env* gene in mink may have been repurposed for host functions.

## 1 Introduction

The retroviruses (RVs) enter host cells by delivering their capsid and viral RNA genomes into the cytoplasm, where viral reverse transcriptase (RT) catalyzes the synthesis of a double-stranded DNA replica of the viral genome. This DNA is then integrated into the host chromosomal DNA. This incorporated DNA becomes an integral part of the genetic material of the infected cell. When integration takes place in germline cells, the virus may pass down through successive generations. Such transmission events can occur in the form of an endogenous viral (or endogenous retroviral; ERV) element (Skirmuntt and Katzourakis, 2019; Barnes and Price, 2023; Liu et al., 2023). ERVs are grouped into the following three categories based on the similarities between their reverse transcriptase (RT) sequences and those of other Retroviridae viruses; class I (gamma- and epsilon-RVs) and class II (alpha-, beta-, delta-, and lenti-RVs) are known as ortho-RVs; class III shows a high similarity with spuma-RVs (Yedavalli et al., 2021). Despite originating from viruses that undergo rapid evolution, ERVs undergo genetic drift following integration into the host genome and are influenced by the neutral mutation rate of the host species (Simpson et al., 2022; Kassiotis, 2023; Dopkins and Nixon, 2024). Throughout the process of evolution, ERVs were not preserved due to the lack of selective forces, leading to their removal or inactivation. As a result, the accumulation of mutations hindered the production of infectious virions, rendering most ERVs inactive (Simpson et al., 2022; Kassiotis, 2023; Dopkins and Nixon, 2024). Occasionally, certain ERV domains are repurposed by their hosts for cellular functions; such sequences are protected from mutational decay by purifying selection, which prevents the deterioration of genetic sequences that occurs under neutral selection (Kassiotis, 2023; Dopkins and Nixon, 2024; Wang et al., 2024).

ERVs exhibit a dual nature in their relationship with present-day infectious RVs. While some ERVs share similarities with contemporary infectious RVs, others are paleovirological remnants of either extinct or yet-to-be-discovered infectious viruses (Diehl et al., 2016; Naville and Volff, 2016; Grandi et al., 2020). Proliferation of available genome sequences has facilitated the documentation of the widespread distribution of ERVs across vertebrates and enabled genome analyses to compile a comprehensive inventory of viral subtypes in various species and trace instances of cross-species transmission (Hayward et al., 2015; Zhuo and Feschotte, 2015; Greenwood et al., 2018; Yedavalli et al., 2021). The discovery of ancient paleo-RVs has prompted researchers to reconstruct ancestral genomes (Blanco-Melo et al., 2018; Yedavalli et al., 2021), which may not have existing infectious counterparts. Although most mammalian ERVs undergo mutational degradation or truncation, there are occasional instances where an ERV gene is advantageous to the host, leading to its incorporation into a physiological function that has been preserved for millions of years because of evolutionary pressures (Johnson, 2019; Simpson et al., 2022). Examples of domesticated genes include viral envelope (*env*) genes that have been repurposed for placental formation and are commonly referred to as syncytins (Dittmar et al., 2021; Bu et al., 2022; Jern and Greenwood, 2024). Additionally, the *env* and *gag* genes have been found to display antiviral capabilities, as exemplified by Fv4 and Fv1 (Ikeda et al., 1985; Best et al., 1996; Ikeda et al., 2001). Furthermore, regulatory sequences that influence host gene expression have been identified (Buzdin et al., 2017; Meyer et al., 2017; Zhou et al., 2021; Plant et al., 2023).

Minks (*Neovison vison*) are one of the most important fur animals; besides providing fur, they are widely used as experimental models in biomedical research. Minks exhibit a yearly pattern of testicular activity, involving shifts between suppressed and highly active spermatogenesis. Thus, they are valuable subjects for investigating the fundamental mechanisms governing spermatogenesis (Blottner et al., 2006; Pelletier et al., 2015). The circannual cycle of minks encompasses three primary stages of testicular function—(i) reactivation of testicular activity in autumn (November), (ii) peak sexual activity (February to March), and (iii) sexual inactivity (May to October) (Zhang et al., 2023).

In this study, we observed upregulation of ERV-derived genes (here named mink ERV-V *env*) during testicular regression. However, the origin and biological function of mink ERV-V *env* remain unclear. A phylogenetic tree was constructed, using Bayesian methods and maximum-likelihood inference, for the mink ERV-V *env* sequences, and reference sequences for both endogenous and exogenous viral envelope genes were included in it. A significant degree of sequence conservation and positive selection was seen within the *env*-surface ORFs in their respective orders; qRT-PCR revealed a wide-ranging expression pattern of mink ERV-V *env* in various tissues. Notably, the expression of mink ERV-V *env* gene in the testicular tissue strongly correlated with their seasonal reproductive cycle. This study would make a significant contribution to the literature because it suggested that the ERV-V *env* gene in mink may have been repurposed to perform different host functions.

## 2 Materials and methods

### 2.1 Ethics statement

The Institutional Animal Care and Use Committee of the Chinese Academy of Agricultural Sciences in Jilin Province, China, reviewed and approved the protocol for animal experiments (Approval number: 2020007) (Zhang et al., 2023). The experiments were conducted in collaboration by the Institute of Special Animal and Plant Sciences, the Chinese Academy of Agricultural Sciences, and Inner Mongolia Agricultural University. Animal testing was done at the Institute of Special Animal and Plant Sciences and sample analysis at Inner Mongolia Agricultural University. Animal testing was carried out following the guidelines of the Animal Research Reporting of *In Vivo* Experiments (ARRIVE).

### 2.2 Sample collection

Male black minks (*Neovison vison*) that were eleven months old were obtained from the research facility at the Chinese Academy of Agricultural Sciences. Each mink was kept in its own cage, given a daily portion of commercial food, exposed to sunlight, and allowed to freely eat and drink (Zhang and Xu, 2022; Zhang et al., 2023).

The minks were euthanized by CO_2_ inhalation. Black mink testes were collected in January (n = 6), February (n = 6), March (n = 6), April (n = 6), May (n = 6), June (n = 6), July (n = 6), August (n = 6), September (n = 6), October (n = 6), November (n = 6), and December (n = 6). The minks used in this experiment were identical to those in Zhang and Xu, 2022 and Zhang et al., 2023.

### 2.3 Isolation and subcloning of Mink ERV-V env cDNA

To construct plasmids expressing C-terminal His-tagged mink ERV-*env*, we used total RNA from mink testes and subjected it to RT-PCR using a Superscript III one-step RT-PCR kit (Invitrogen, Grand Island, NY). The RT-PCR program was as follows: incubation at 55°C for 30 min, denaturation at 95°C for 3 min, 35 cycles of denaturation at 95°C for 30 s, annealing at 60°C for 30 s, and extension at 72°C for 2 min, followed by a final extension step at 72°C for 10 min. The relevant primers (F: 5’- CGCGGATCCCGGCCATGATGAGACTGATGAAGTGGAC -3,’ R: 5’- CGCGAATTCCGTGAACCTCCACCTCCTGAACCTCCACCTCCGGTAGAGAGCCTCTGCCTGCGTC-3’) were used for RT-PCR. The resulting PCR products were purified using a QIAquick gel extraction kit (QIAGEN, Shanghai, China), and the mink-ERV-V*-env* ORFs were cloned into the pCDNA4/Myc-His plasmid using EcoRI (Thermo Fisher Scientific, Shanghai, China) and EcoRV (Thermo Fisher Scientific, Shanghai, China) restriction enzymes.

### 2.4 Quantitative reverse transcription-PCR amplification

In accordance with the manufacturer’s instructions, an RNeasy Mini Extraction Kit and RNase-free DNase Set (Qiagen, Shanghai, China) were used to extract total RNA. The first strand of cDNA was synthesized from 2.5 μg of total RNA using SuperScript III (Invitrogen, Grand Island, NY) primed with oligo dT. All qRT-PCR reactions were performed using 2×SYBR Green qPCR Master Mix (Life Technologies, Grand Island, NY). All qRT-PCR analyses were performed using Roche LightCyler 480 equipment with a 20-μL reaction volume. The RT-PCR primers used were *env*-F (5’- TAGGAGAGCTCTTGGGCGTT-3’) and *env*-R (5’-TCCTGGCGGCTCTCTAAACT-3’). Relative expression of the selected genes was adjusted using *GAPDH* as an endogenous control (Zhang et al., 2023). The study employed the comparative cycle threshold method to determine the amplification fold, with the cycle threshold (Ct) representing the number of PCR cycles needed to reach the fluorescence threshold. The calculation of ΔCt involved subtracting the Ct value of the reference gene GAPDH from that of the target gene. The fold change was calculated as 2^–ΔΔCt^, where ΔΔCt is defined as the difference between the ΔCt values of the sample and control groups. Each time point, three testes were collected from three different animals, each providing one RNA sample.

### 2.5 Database search for mink ERV-V env orthologs

ORF sequences from mink ERV-*env* were utilized as probes in a BLAST query of 66 Carnivora genome assemblies archived in the GenBank repository. A BLASTn query was performed with gap costs set at 5 and 2 for existence and extension, match/mismatch scores of +2/−3, repeat masking filter turned off, and an expect threshold of 10^−75^ established.

### 2.6 Phylogenetic analysis

We examined the genomic area encompassing BTN1A1 and BTN2A1 as well as the regions extending 150 kb past SPTLC3 to study synteny in Carnivora for ERV-*env* and associated ERV duplicates (Simpson et al., 2022). The segments were obtained from the latest GenBank assembly for each species (**Supplementary Table S1**), and a sequence alignment was performed using MultiPipMaker (Chen et al., 2017; Horie and Tomonaga, 2019; Blanco-Melo et al., 2024).

Phylogenetic trees were built using the neighbor-joining method in MEGA-X (Kumar et al., 2018). Two trees were constructed using the RT domain of pol and a portion of the transmembrane subunit of the *env* gene. **Supplementary Table S2** shows the ERV sequences utilized for tree construction. The JTT matrix-based method (Kumar et al., 2018) was used to compute evolutionary distances, and a gamma distribution (shape parameter = 1) was employed to model the rate of variation across sites. Positions with site coverage below 95% were removed to ensure less than 5% alignment gaps, missing data, or ambiguous bases at any position (Langmead and Salzberg, 2012; Alser et al., 2021; Warnow, 2021). The amino acid sequences of mink ERV-V *env* were aligned using the MUSCLE algorithm within Geneious Prime with default parameters. Maximum-likelihood phylogenetic trees were constructed using the RaxML program with the General Time Reversible + G + I model and 500 bootstrap replicates for branch support.

### 2.7 Bioinformatic analysis

The JPred secondary structure prediction server (Drozdetskiy et al., 2015) was utilized to query the amino acid sequences of mink ERV-V Env and human EnvV2. The resulting JNetPRED and JNetCONF graphs were extracted and analyzed to determine their secondary structures. ProtScale was used to plot the hydropathicity scores for these proteins (https://web.expasy.org/protscale/).

### 2.8 Homology modeling

The mink ERV-V Env sequence was analyzed using the I-TASSER (Yang et al., 2015) program, which employs a multiple-threading approach to identify homologs. The approach involves identifying templates in the Protein Data Bank (PDB), conducting iterative structure-assembly simulations, selecting and refining models, and annotating structure-based functions.

### 2.9 Cell culture, transfection, western blotting, and immunofluorescence

Cells (293T) were grown in a 12-well dish with 200,000 cells per well and then transfected with either 1000 ng pcDNA4-mink-ERV-*env*-His or pcDNA4-empty plasmids using Lipo8000™ Transfection Reagent (Beyotime Biotechnology, Shanghai, China). Cells were lysed with RIPA buffer (Beyotime Biotechnology, Shanghai, China) after three days using the same number of cells. Equal volumes of lysates were loaded onto a 4–12% Bis-Tris polyacrylamide gel for SDS-PAGE analysis. Immunoblot analysis was conducted on a PVDF membrane, and the results were captured with a Tanon 5200 imaging system from Tanon (Shanghai, China). The following antibodies were used for protein detection: Anti-6× His tag antibody (Cat. No. ab18184; Abcam Plc., Cambridge, UK); anti-GAPDH antibody (Cat. No. 60004–1-Ig, Proteintech Group Inc., Wuhan, China); Goat Anti-Rabbit IgG H&L (HRP) (Cat. No. ab205718; Abcam Plc., Cambridge, UK).

Cultured mink testis cells on 25-mm coverslips were transfected with 200 ng of either pCDNA4/Myc-His or pcDNA4-mink-ERV-*env*-His plasmid in 12-well plates. We fixed the cells with 3.7% formaldehyde and permeabilized them with PBS containing 0.1% Triton X-100 after two days. Primary mouse anti-His antibody (Cat. No. ab18184; Abcam Plc., Cambridge, UK) was added to 10 mM PBS containing 10% normal goat serum. After three washes with PBS, the sections were exposed for 30 minutes to secondary antibodies (Goat Anti-Mouse IgG H&L (Alexa Fluor^®^ 647); Cat. No. ab150115; Abcam Plc., Cambridge, UK) diluted in PBS. In negative controls, primary antibody is PBS. Cell nuclei were stained with DAPI (Beyotime Biotechnology, Shanghai, China) and then placed on glass slides using the ProLong antifade kit (Thermo Fisher Scientific) for observation under a Leica fluorescence microscope DM2500.

### 2.10 Statistical analysis

A one-way ANOVA analysis of the data was performed with the PRISM software, and then Tukey’s multiple comparison test was conducted using GraphPad Software, Inc. The findings are displayed as the average ± standard error of the mean of values from a minimum of three biological duplicates. Statistical significance was set at a p-value of less than 0.05.

## 3 Results

### 3.1 Differential expression of mink ERV-V env associated with total testicular regression and recrudescence in mink

**Figure 1:**
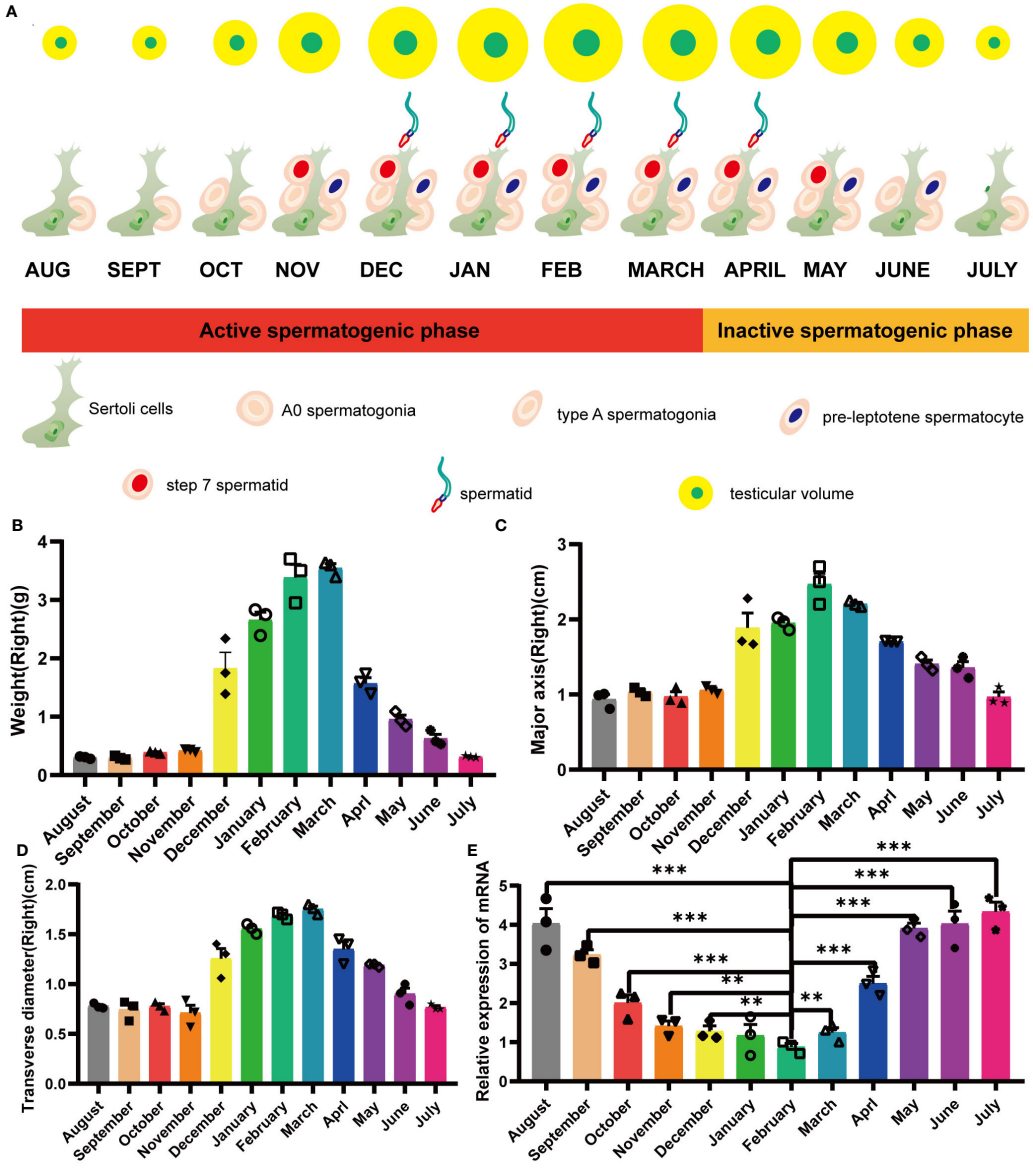
Yearly seasonal breeding cycle in a typical mature mink. **(A)** Cellular changes in the testes during the annual seasonal reproductive cycle. **(B)** Variation in mink testis weights during the annual seasonal breeding cycle. **(C)** Variation in mink testis major axis during the annual seasonal breeding cycle. **(D)** Variation in mink testis transverse diameter during the annual seasonal breeding cycle. **(E)** ERV-*env* expression in normal adult mink testes during the annual seasonal reproductive cycle. A one-way ANOVA was employed to determine significant differences between the different time points (*p < 0.05; **p < 0.01; ***p < 0.001).

Male minks of seasonal brooding breeds experienced a decrease in seminiferous tubule diameter and a consequent decrease in testis size during testis regression (**Figures 1A–D**). In minks, the testes underwent significant circannual changes in both structure and function, involving the elimination of the germinative epithelium during the non-breeding period (**Supplementary Figure S1**). This phenomenon resulted in notable alterations in the processes and structures, such as androgen production, apoptosis, cell proliferation, and integrity of the blood-testis barrier (BTB) (Zhang et al., 2023). In a previous transcriptomic study that examined mink testes at different reproductive stages (active, regressing, and inactive), we identified an interesting gene (specifically, an endogenous retrovirus *env* gene) and named it mink ERV-*env*. In the current study, we found that variation in expression of this gene was highly consistent with testis regression. Quantitative PCR results revealed that its expression was elevated during testis regression in minks, remained at high levels during the non-breeding season, decreased during testis recrudescence, and was maintained at low levels during the breeding season (**Figure 1E**).

### 3.2 Conserved env ORF in carnivores related to the mink ERV-V env ORF

**Figure 2:**
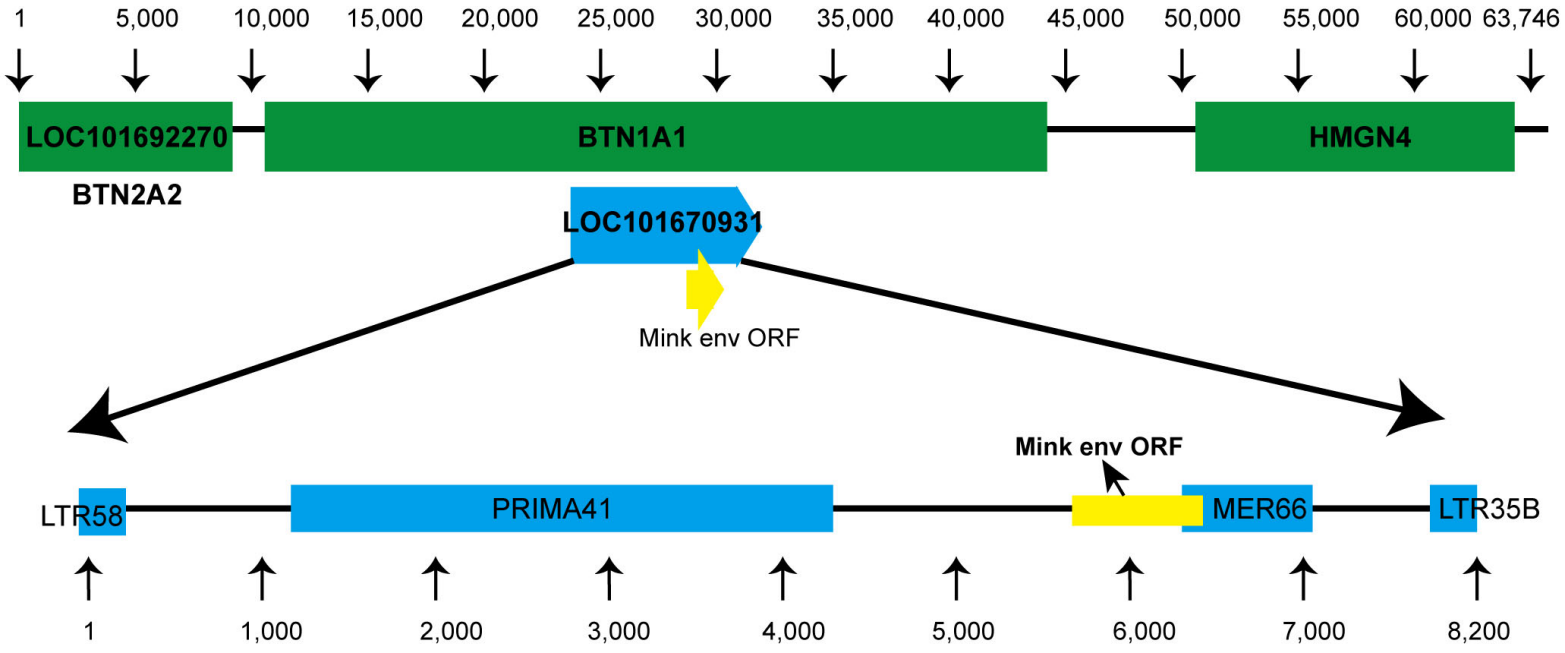
Genomic location of mink ERV-*env*. Location of the mink ERV-*env* gene is illustrated on chromosome 1 (NC_058091.1) in the genome of *Neovison vison* (mink). Green indicates RefSeq annotated genes, while yellow represents the mink ERV-*env* ORF. Blue annotations indicate ERV-derived repetitive elements that were identified in the specified region based on data in the Dfam repeat database.

Using tBLASTn, we analyzed annotated mammalian genomes in-silico to discover ERVs associated with mink ERV-*env*. As anticipated, the majority of matches corresponded to *env*V orthologs in Carnivora. A more detailed analysis of the genetic location of this ERV in the mink species *Neovison vison* showed that it is situated between the *BTN1A1* and *BTN2A1* genes (**Figure 2**). Furthermore, a coding region was identified at the 3′ terminus of this ERV, located before the expected 3′ long terminal repeat (LTR) (**Figure 2**). Further analysis of mink ERV-V in the Dfam repeat database uncovered remnants of the *gag* and *pol* genes that resembled an ERV cluster known as Prima41, whereas the C-terminal portion showed similarities to the ERV cluster MER66 (**Figure 2**).

**Figure 3:**
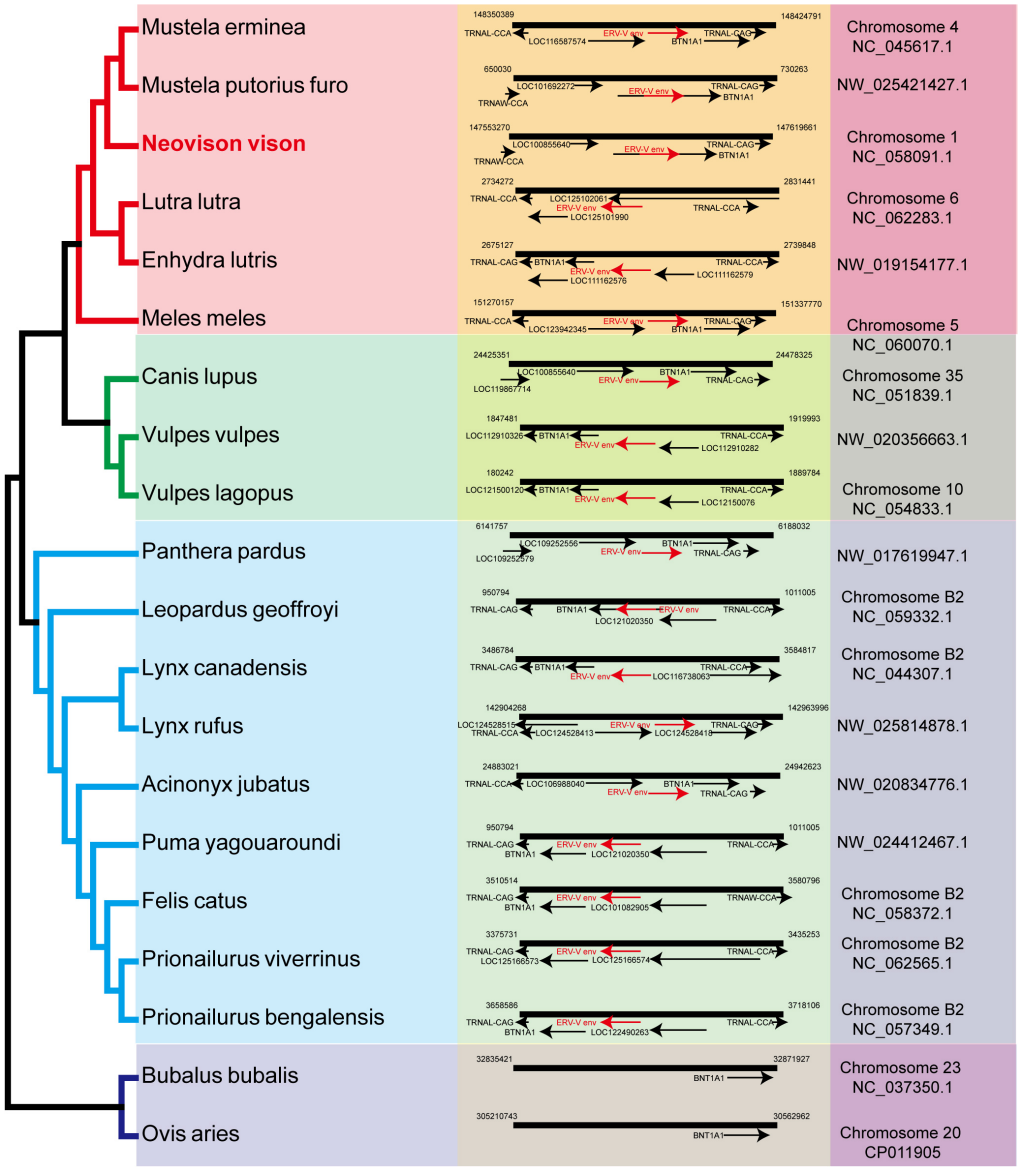
Genomic synteny of mink ERV-*env*. Genomic segments containing the mink ERV-*env* ORF and *BTN1A1* and *BTN2A1* genes were extracted from the NCBI genome database for each of the indicated species and aligned. The mink ERV-*env* ORF, along with the BTN1A1 and BTN2A1 exons, are highlighted in red and black arrows in the reference assembly.

**Figure 4:**
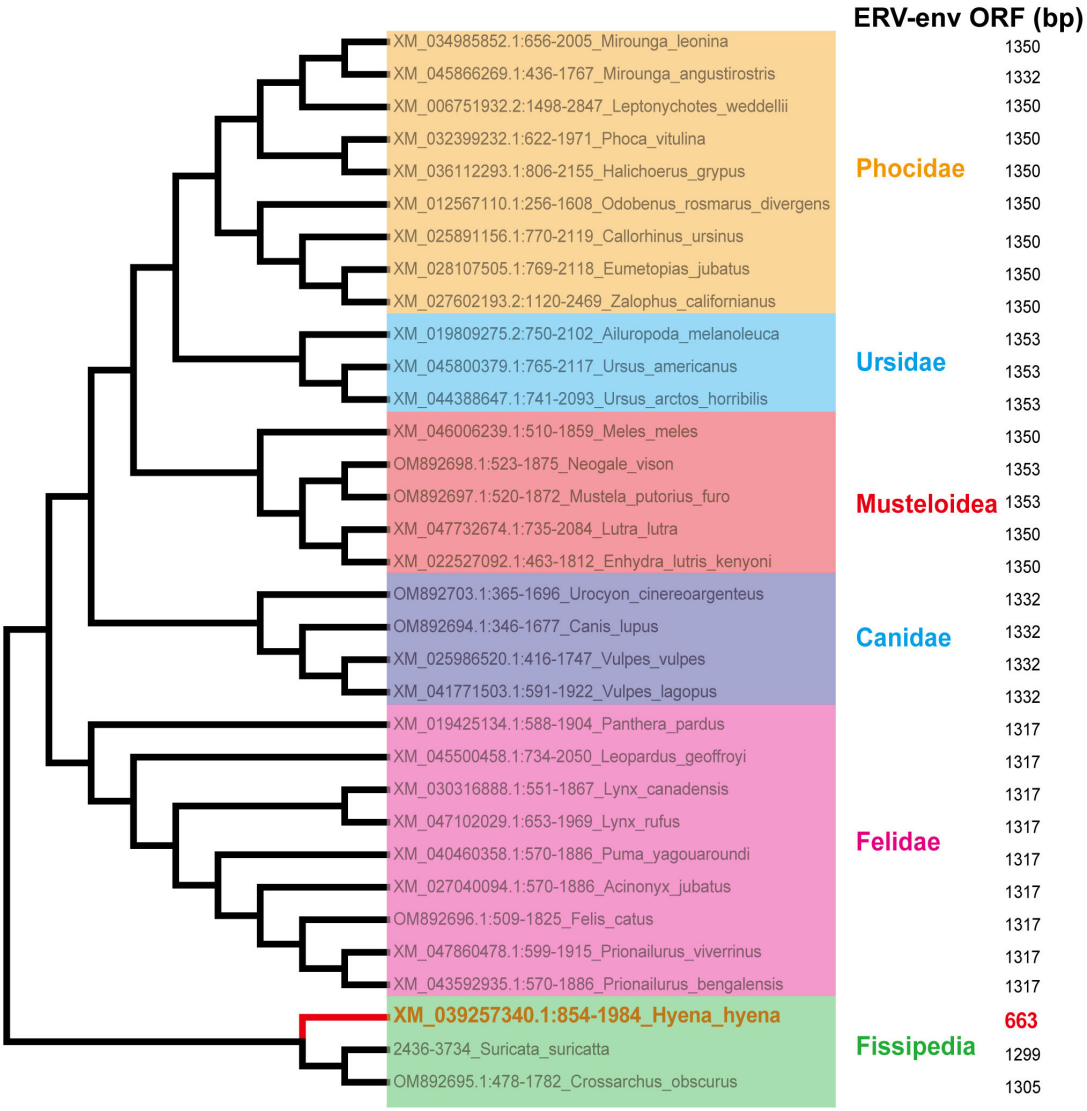
Conservation of mink ERV-*env* ORF in Carnivora lineages. Mink ERV-env-containing ERV orthologs were identified through BLAST searches of individual genome assemblies. MEGA v7.0.26 was used to create this cladogram, which shows the evolutionary connections among species with complete genomes in the order Carnivora. Classifications of suborder and family are displayed on the right side.

Sections of the mink ERV-V *env* ORF were retrieved and compared with homologous sequences from 18 Carnivora species and two closely related species in the superorder Laurasiatheria (**Supplementary Table S1**). The mink ERV-V *env* ORF showed significant sequence similarity across Carnivora, with the majority of exons in the associated *BTN1A1* and *BTN2A1* genes in all species analyzed resembling one another, thereby confirming the orthologous nature of this region (see **Figure 3**). Furthermore, the region encompassing the mink ERV-V *env* ORF, along with the rest of the provirus, was found to be absent in all mammalian species other than those in Carnivora (**Figure 3**). The mink ERV-V *env* gene, with its long ORF (**Figure 4**), is preserved in all Carnivora lineages except Hyaenidae, where premature stop codons in the orthologous gene lead to the formation of shortened ORFs (**Figure 4**).

In a manner akin to host genes, ERV genes, once integrated and stabilized within a lineage, may undergo conservation through purifying/negative selection to maintain advantageous functionality, reflected by a dN/dS (ω) ratio below 1, or alternatively, they may experience evolutionary changes through diversifying/positive selection, leading to adaptive modifications and a ω value exceeding 1. To discern the indications of selection pressures within mink ERV-V *env* gene, we employed diverse analytical approaches available on the Datamonkey web server. Three residues (133R, 437D, 448R) within the mink ERV-V *env* were identified as undergoing positive selection by the MEME and FEL programs on the Datamonkey webserver. While the orthologs of mink ERV-V *env* displayed high sequence similarity at most sites, the pairwise ω values were predominantly below unity across the majority of species. These findings suggest that mink ERV-V *env* have experienced repeated episodes of positive selection within their respective orders.

### 3.3 Identification of the mink ERV-V proviruses as gamma-like ERVs

**Figure 5:**
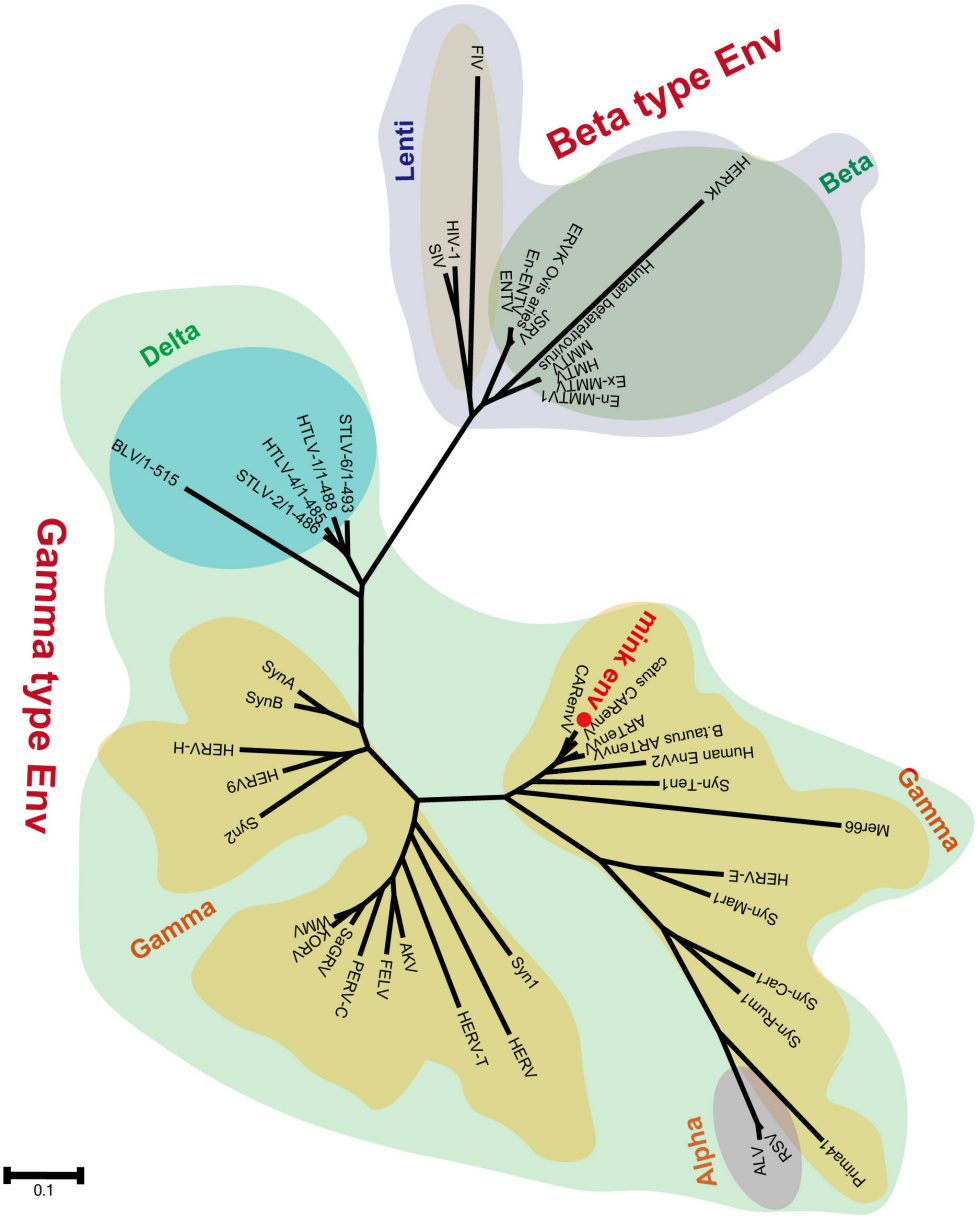
Phylogeny of mink ERV-env protein. The predicted amino acid sequence of mink-ERV-env (highlighted in red) was juxtaposed with the amino acid sequences of selected endogenous and exogenous retroviral Env proteins, leading to the construction of a maximum likelihood tree with 500 bootstraps in RaxML.

**Figure 6:**
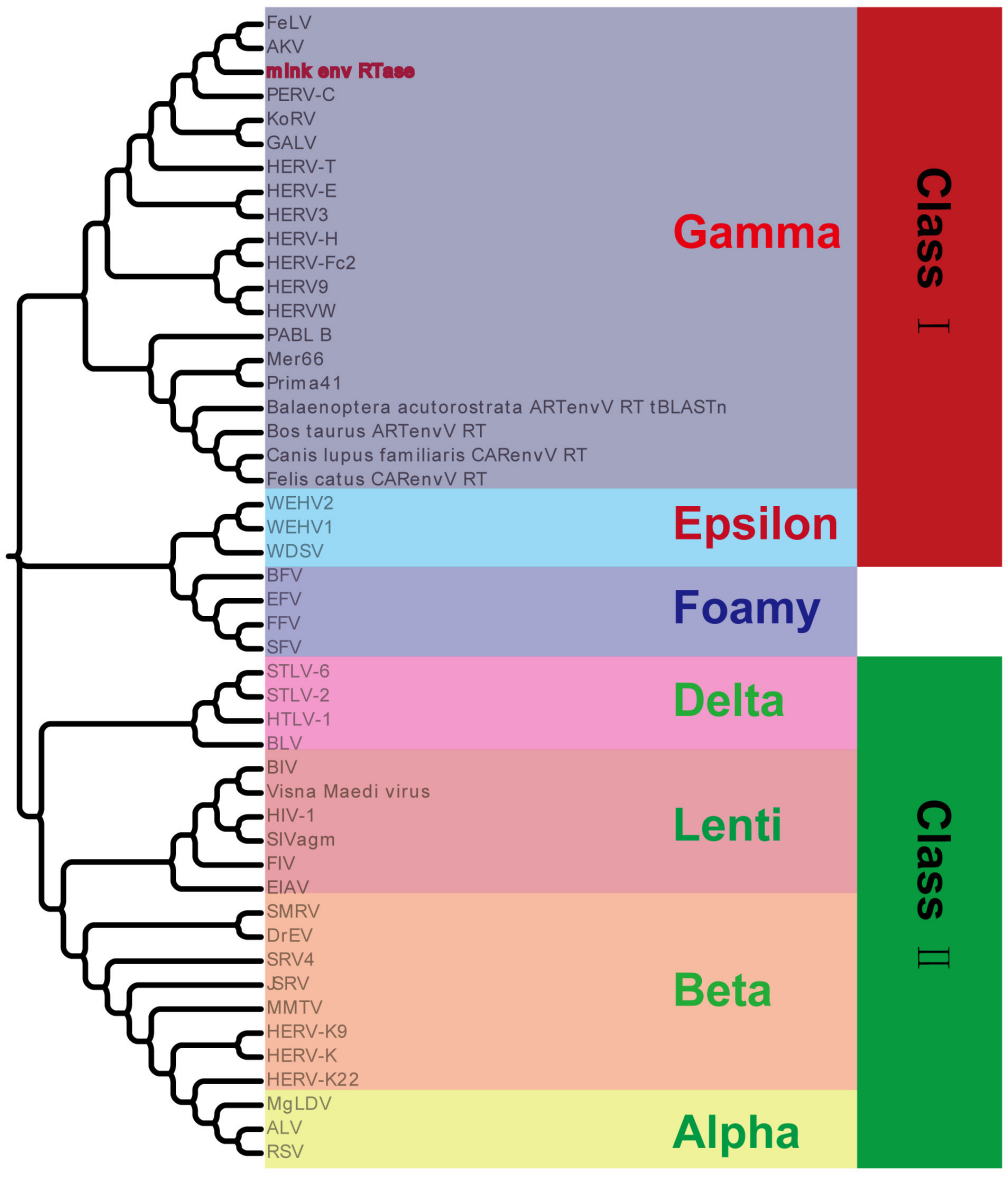
Phylogeny of mink ERV RT protein. Mink-ERV RTs were aligned with those of endogenous and exogenous RVs from different species, and a maximum likelihood tree was constructed with 500 bootstraps using RaxML.

Segments of retroviral genomes can exhibit diverse phylogenetic histories because of frequent retroviral recombination, which can involve distantly related RVs (Henzy et al., 2014; Yedavalli et al., 2021) or occur between endogenous and exogenous viruses (Bamunusinghe et al., 2017). RVs are categorized into two subfamilies, namely orthoretroviruses and spumaretroviruses (Simpson et al., 2022). Orthoretroviruses are further classified into six genera, namely *Alpharetrovirus*, *Betaretrovirus*, *Gammaretrovirus*, *Epsilonretrovirus*, *Lentivirus*, and *Deltaretrovirus* (Simpson et al., 2022). ERVs are categorized into three classes—class I (gamma and epsilon), class II (alpha, beta, delta, and lenti), and class III (spuma)— depending on how closely they resemble exogenous RVs (Gifford et al., 2018). Retroviral envelope proteins can be classified into two subtypes, namely gamma-type and beta-type, depending on the linkage type (covalent or non-covalent) between the SU and TM subunits (Henzy and Johnson, 2013). In the phylogenetic analysis of both endogenous and exogenous retroviral envelope proteins, mink ERV-V Env proteins grouped together with those from the gamma-type envelope subgroup, which has SU and TM subunits linked together covalently. This subgroup encompassed gamma, delta, and alpha RVs (**Figure 5**). Mink ERV-V *env*, CARenvV, and ARTenvV formed a group with human EnvV2 (**Figure 5**). By comparing amino acid sequences of RVs from different sources, including those of mink ERV-V ERVs, we created a phylogenetic tree to classify retroviral groups. All seven RV genera had highly conserved RT cores, and the tree displayed three groups corresponding to ERV classes I–III (**Figure 6**). **Figure 6** shows that the RT from mink ERV-V ERVs grouped together with external gammaretroviruses and early mammalian gamma-like class I ERVs such as FeLV, PERV-C, and AKV.

### 3.4 Prediction of conserved domains and functional classification of mink ERV-V ENV protein

**Figure 7:**
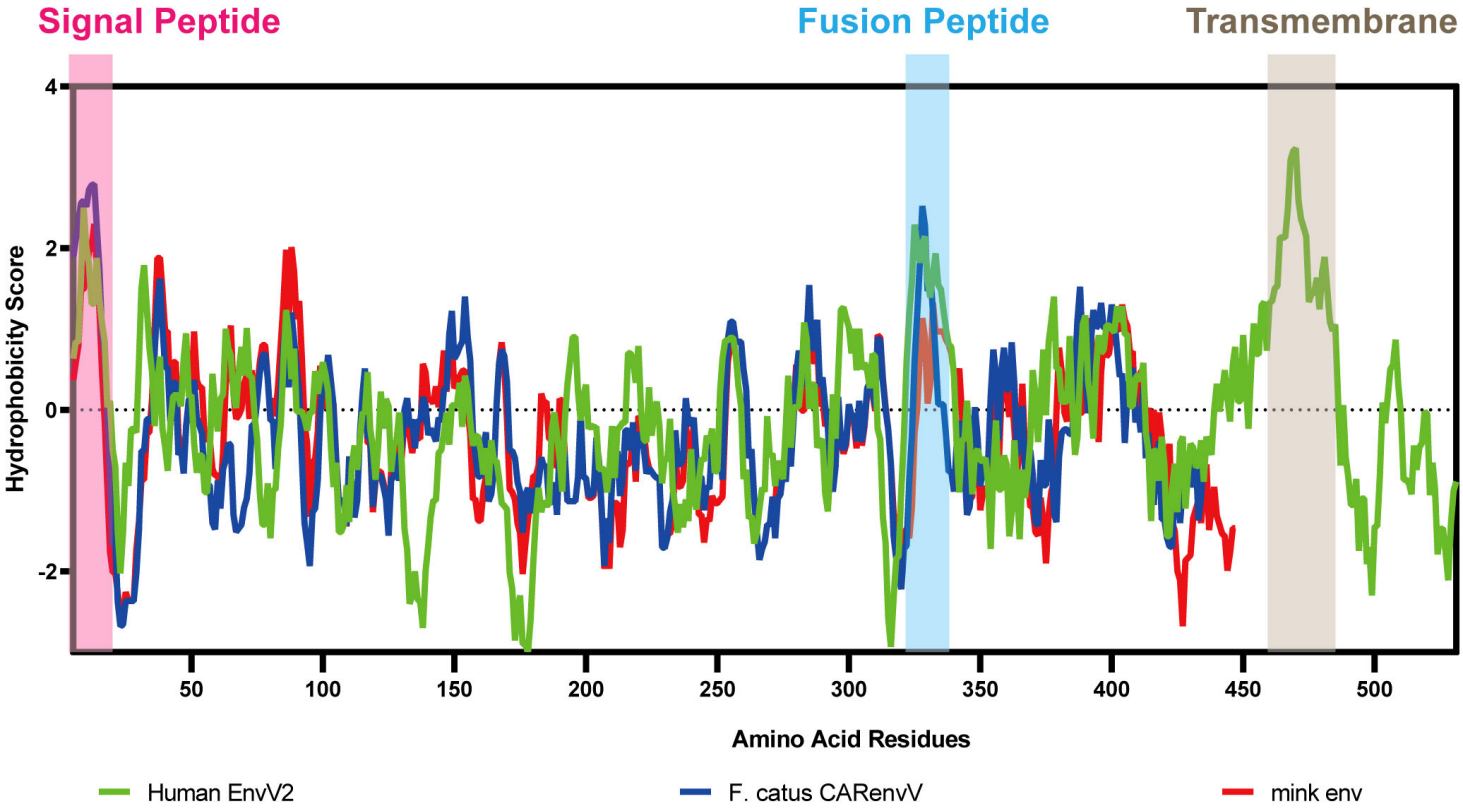
Hydrophobicity profiles are shown for human EnvV2 (green), *Felis catus* CARenvV (blue), and mink ERV-V ENV (red).

**Figure 8:**
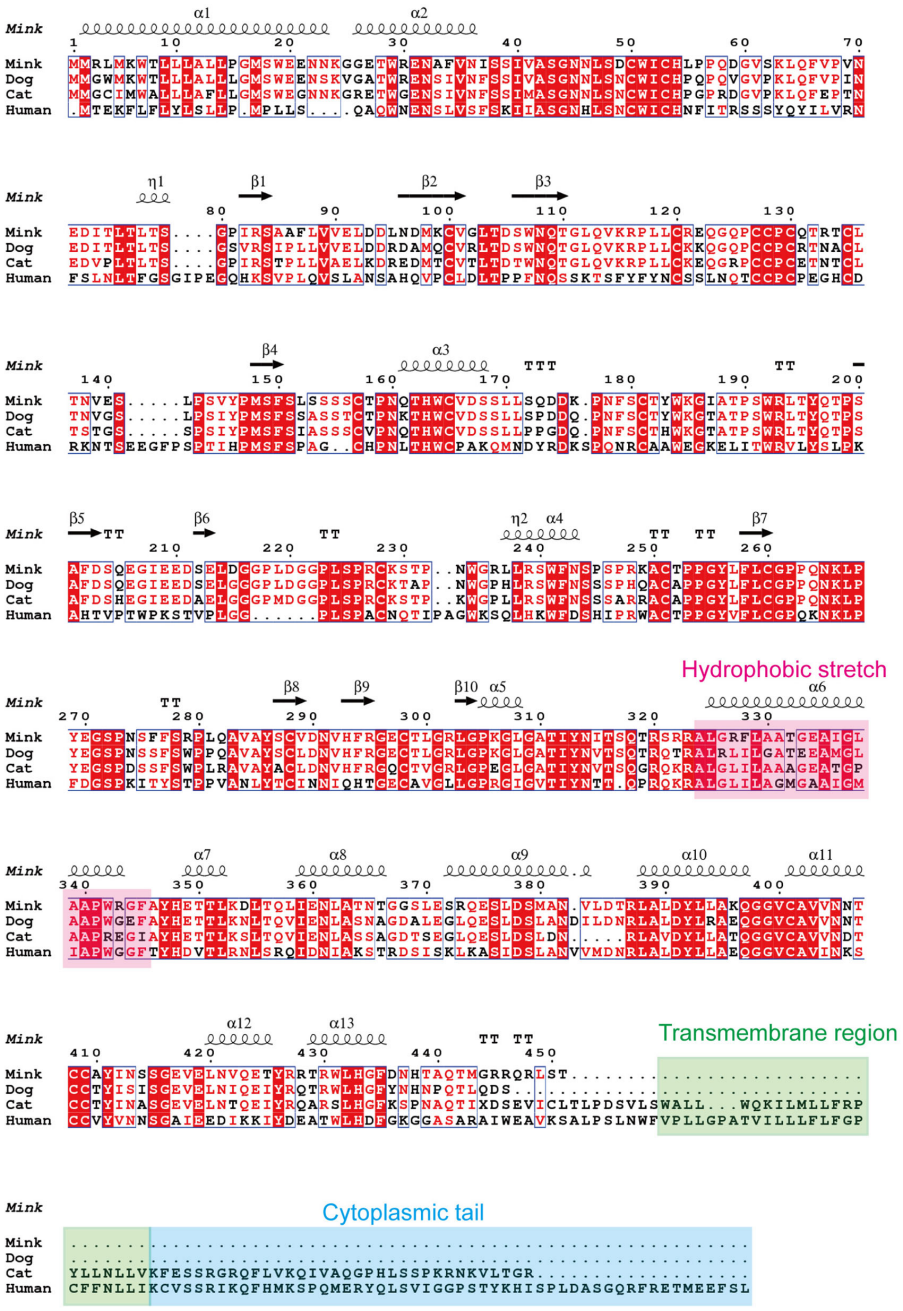
Secondary structure predictions are shown for human EnvV2, cat CARenvV, dog CARenvV, and mink ERV-V Env. In secondary structure prediction, whorl-like structures indicate alpha helices and arrows indicate beta sheets.

To ascertain the presence of retroviral Env protein characteristics in the mink ERV-V Env protein, hydrophobicity plots were generated for the protein with human EnvV2. In addition, predicted secondary structures of these proteins were generated. The results of our analysis suggested the presence of a possible N-terminal signal peptide containing both a hydrophobic area and an alpha helix. Additionally, a potential fusion peptide was discovered in the C-terminus that contained both a hydrophobic section and an alpha helix (see **Figures 7**, **8**). A comparison of the amino acid sequences of human EnvV2 and mink ERV-V Env protein showed that no full transmembrane region was present.

**Figure 9:**
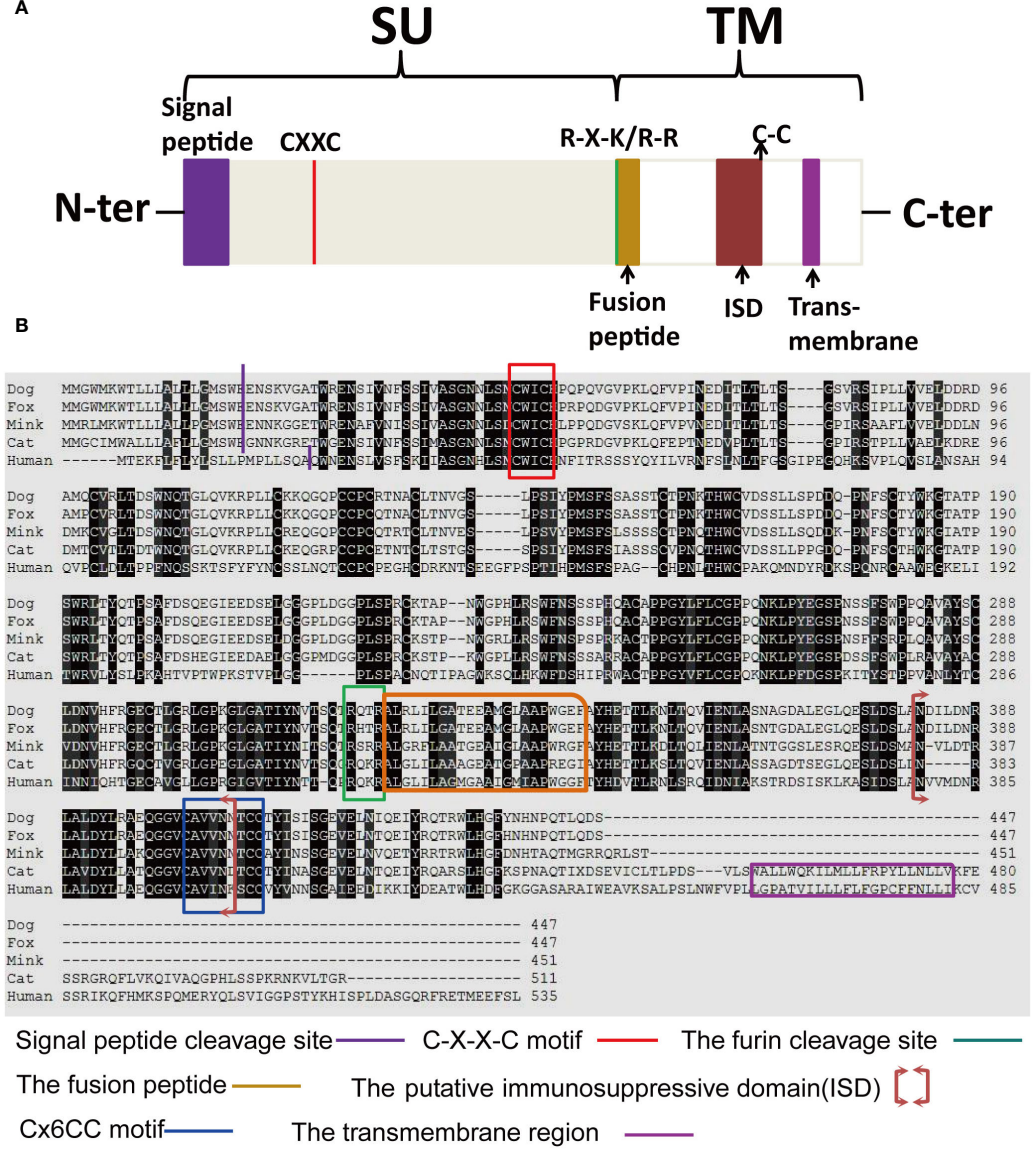
Structural features of the mink ERV-V Env protein. **(A)** Protein domain pattern map of mink ERV-V Env. **(B)** Comparison of the amino acid sequences of human EnvV2 with those of ERV-V Env from dog, cat, fox, and mink. The purple vertical line indicates the signal peptide cleavage site. The red square indicates the CXXC motifs that are involved in SU-TM interactions. The furin cleavage site (RXXR) is denoted by the green square. The pink square indicates the fusion peptide. The brown square brackets indicate the putative immunosuppressive domain (ISD). The green square indicates a Cx6CC motif. The purple square indicates the transmembrane domain (TM). Black shading indicates the residues that are identical in all proteins, while different shades of grey background indicate the residues that are identical in four, three, or two proteins.

The structure and characteristics of the canonical retroviral Env protein are shown in **Figure 9A**. Human EnvV2 showed the most resemblance with these Env proteins in the area right after the expected signal peptide (**Figure 9B**). The mink ERV-V Env protein was truncated immediately before the membrane-spanning domain, which was located close to the Env C-terminus (**Figure 9B**). The alignment revealed two conserved cysteine-containing motifs in the mink ERV-V Env protein, CxxC in the SU domain, and Cx6CC in the transmembrane domain, which were present at the same location as in EnvV2 (**Figure 9B**). The mink ERV-V Env protein contained the RxxR furin cleavage motif (RQT/SR) immediately before the anticipated fusion peptide (**Figure 9B**). The immunosuppressive domain and heptad repeats found in the mink ERV-V Env protein are similar to those found in the previously discovered EnvV2 immunosuppressive domain and heptad repeats (**Figure 9B**).

### 3.5 Structural analysis of the protein coded by the mink ERV-V Env gene

**Figure 10:**
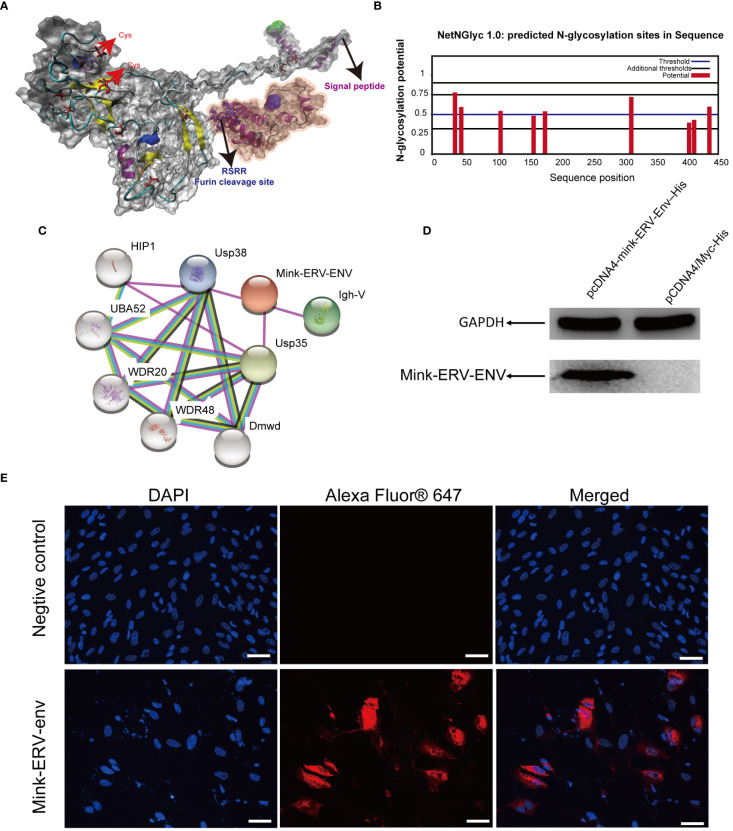
Comparative homology modeling and functional analysis of the mink ERV-V Env protein. **(A)** Three-dimensional ab-initio models of mink ERV-V Env proteins. **(B)** Post-translationally modified glycation sites in the mink ERV-V Env protein. **(C)** Map of protein-protein interactions with the mink ERV-V Env protein. **(D)** Western blot examination of His-tagged mink ERV-V Env protein in 293T cells. Lane 1, a pcDNA4-mink-ERV-Env–His plasmid was transfected into 293T cells. Lane 2, a pCDNA4/Myc-His plasmid was transfected into 293T cells. **(E)** Confocal microscopic examination of His-tagged mink ERV-V Env protein in mink testis cells shows accumulation in the cytoplasm (scale bar, 100 μm).

We next modeled the three-dimensional structure of the mink ERV-V Env protein. Five models were created on the I-TASSER web server, and the best model was selected and visualized using PyMOL (**Figure 10A**). The possible signal peptide and furin cleavage motif of mink ERV-V Env are shown in **Figure 10A**. Attempts were made to predict the protein modification sites in mink ERV-V Env; results indicated six potential N-glycosylation sites in the disordered region of mink ERV-V Env at amino acid positions 36 (NISS), 46 (NLSD), 108 (NQTG), 178 (NFSC), 314 (NITS), and 437 (NHTA) (**Figure 10B**). The STRING database v12.0 was used to study the interactions between mink ERV-V Env and other proteins (**Figure 10C**). Data from the STRING database indicated that interactions between mink ERV-V Env and ubiquitin carboxyl-terminal hydrolase 35 (USP35), USP38, and Ig-like domain-containing protein. In transfected 293T cells, the mink ERV-V Env protein labeled with histidine showed consistent expression when inserted under the constitutive CMV promoter (**Figure 10D**). The aforementioned expression plasmids were transfected into the primary testicular cells of minks, which resulted in the accumulation of this protein in the cytoplasm (**Figure 10E**). Importantly, the Env protein from different RVs was found in the cytoplasm during infection, but as the viral life cycle progressed, this protein localized to the plasma membrane for assembly. A major reason for this is that the mink ERV-V Env protein has lost its transmembrane region, which prevents it from being expressed at the cytoplasmic membrane.

### 3.6 Mink ERV-V env gene expressed in various tissues

**Figure 11:**
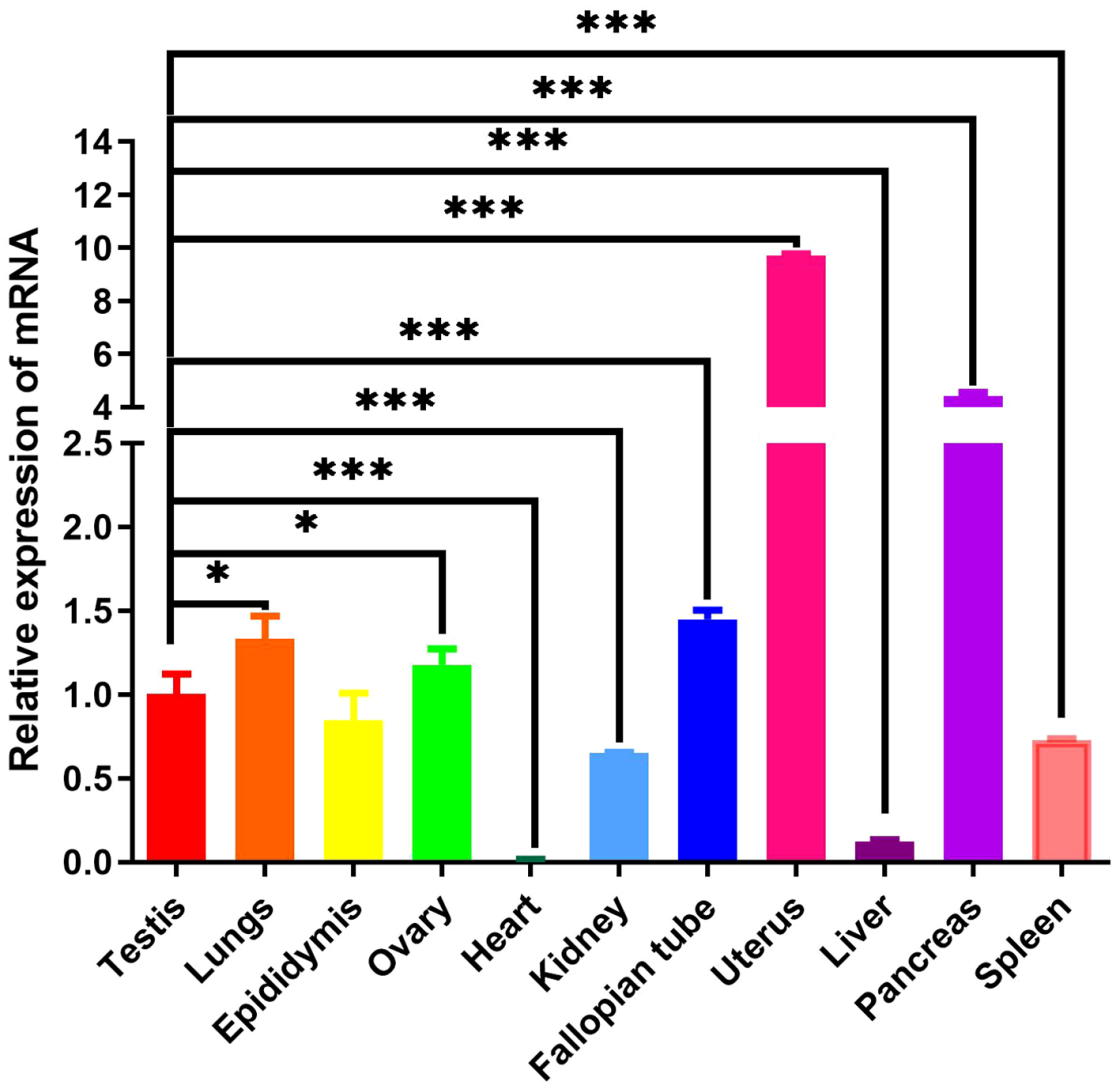
Mink ERV-Env gene is expressed in a wide variety of tissues. A one-way ANOVA was employed to determine significant differences between the different tissue samples (*p < 0.05; **p < 0.01; ***p < 0.001).

To ascertain the tissue-specific expression profile of mink ERV-V Env gene, we employed RT-qPCR. GAPDH was utilized as the internal reference gene. Mink ERV-V Env gene exhibited varying expression levels across a diverse range of tissues (Tissues from minks were collected in February), as shown in **Figure 11**. While reduced expression levels of mink ERV-V *env* gene were observed in the heart, liver, kidney, and spleen, increased expression was observed in the uterus and pancreas.

## 4 Discussion

Seasonal breeding refers to the absence of continuous reproductive activity throughout the year in specific species or populations residing in non-equatorial regions of the Earth that occur as a consequence of cyclical environmental variations caused by climatic seasons. In females, ovulation is suspended during a specific period, while in males, testicular function is diminished or exhausted during the non-breeding season (Beltran-Frutos et al., 2022; Gumulka et al., 2022; Tao et al., 2022; Pandey and Mohanty, 2023; Zhang et al., 2023). In seasonal breeding animals such as minks, testes undergo cyclical activation and inactivation annually, which is a particularly interesting example of spermatogenesis regulation (Zhang et al., 2023). In this study, we identified a novel gene, mink ERV-V *env*, which showed expression in testes that was accompanied by cyclic changes in spermatogenesis. The expression of mink ERV-V env gradually increased during the regression stage of mink testes and gradually declined during the transition to the reproductive stage.

In this study, we identified ERVs that are related to mink ERV-V env, the majority of which correspond to Carnivora envV orthologs. The sequences likely represent ancient coding sequences that have been preserved. Endogenous viruses evolve at the host’s mutation rate rather than at the rate of their viral progenitors once they enter the host genome through vertical transfer (Skirmuntt and Katzourakis, 2019). This phenomenon gives rise to two potential outcomes for such sequences, namely the accumulation of mutations, which results in partial loss of the coding sequence and gradual deterioration of its function if it proves detrimental or non-beneficial for the host’s survival, or fixation and preservation of these sequences within the genomes of the host population if they confer a selective advantage (Feschotte and Gilbert, 2012; Skirmuntt and Katzourakis, 2019). The observation of numerous viral sequences in ERVs associated with Carnivora envV orthologs indicates that these integrations occurred in the distant past and have since been deactivated and undergone decay. A small number of species developed premature stop signals in ORFs homologous to mink ERV-V *env*, which are preserved in every lineage across orders. Alignment and phylogenetic analyses of ERV-V *env* sequences will not be affected by this premature stop signal.

In our evolutionary tree, we noticed that mink ERV-V Env proteins grouped together with those from the gamma-type envelope subgroup, known for having surface and transmembrane subunits linked by covalent bonds. Various vertebrate species, including species of mammals, birds, reptiles, and amphibians, harbor ERV loci related to *Gammaretrovirus* (Gifford et al., 2018; Hogan and Johnson, 2023). Overall, Gammaretrovirus-related ERV represent a “fossil” record of the history of retroviruses. This study revealed that mink ERV-V Env contains a possible N-terminal signal peptide that includes both a hydrophobic area and an alpha helix. Additionally, a fusion peptide was predicted in the C-terminus, which contained a hydrophobic segment and an alpha helix. Although mink ERV-V Env lost the transmembrane region and the cytoplasmic tail. But the mink ERV-V Env TM domain has one hydrophobic stretch that flanks an ectodomain. The latter contains two heptad repeats (hr1 and hr2) flanked by two or three cysteine residues (Henzy and Johnson, 2013). Heptad repeats are of utmost importance in facilitating dynamic rearrangement of the trimer during the fusion process as well as in the formation of the widely conserved coiled-coil structure observed in numerous viral fusion proteins (Chen et al., 2022). Additionally, the ectodomain sequence of the mink ERV-V Env transmembrane subunit encompasses a segment referred to as the immunosuppressive domain, which consists of 20 amino acids directly preceding the initial cysteine residue and is distinguished by its conserved residues.

Envelope glycoproteins of the gamma type undergo post-translational modifications such as glycosylation and furin-mediated cleavage, similar to the envelope glycoproteins of other RVs. Gamma-type Envs are estimated to have 4–13 glycosylation sites, the majority of which are located in the surface subunit of the protein. Our study identified six potential N-glycosylation sites in the disordered region of the mink ERV-V Env surface subunit. While there was significant variation in the glycosylation sites, there was far more conservation in the furin cleavage sites. The mink ERV-V Env protein also contains an RxxR furin cleavage motif (RQT/SR) immediately before the anticipated fusion peptide.

Our study results indicate that the mink ERV-V Env mRNA is present in different tissues, and the ORF of this gene can reliably produce proteins when activated by a constitutively active promoter. However, the underlying reasons for the conservation of these retroviral genes remain unclear. It is important to note that ERV loci in vertebrate genetic material are subject to natural selection, with increasing evidence showing the incorporation of ERV-produced proteins during the evolution of host cells and organisms for various functions (Malfavon-Borja and Feschotte, 2015; Frank and Feschotte, 2017; Johnson, 2019). For instance, *Gammaretrovirus*-related ERVs encompass placental syncytin genes that have been evolutionarily co-opted by various mammalian lineages (Zhang et al., 2015). In addition, specific endogenous retroviral genes have been utilized throughout host evolution to provide protection against exogenous retroviruses (Helmy and Selvarajoo, 2021; Chen et al., 2022; Miyake et al., 2022). In addition to the presence of syncytin genes and receptor interference, which originated tens of millions of years ago, there are many cases of ERV loci with intact gamma-type Env ORFs of unknown purpose (Grandi et al., 2020; McCarthy et al., 2020; Simpson et al., 2022; Hogan and Johnson, 2023), with some of them remaining unchanged for more than 100 million years (Kasperek et al., 2022). The extensive tissue expression of mink ERV-V *env* genes suggests that they may have more than just placenta-specific roles (Funk et al., 2019). It is possible, however, that these genes interfere with RVs that bind the same receptor, as shown by certain genes found in mice and cats that are derived from ERV env (Ito et al., 2013, Ito et al., 2016). To explore these possibilities, further investigation into the tertiary structure of mink ERV-V Env and specific cell surface receptors for each protein would be needed. Furthermore, investigation of mink ERV-V Env ORF expression was accomplished by expressing tagged versions of the protein; however, the use of specific antibodies in primary cells from related species may reveal more about its natural expression levels and its location within the cell. In the future, investigation of the role of mink ERV-V Env in testicular regression is warranted.

**Figure 12:**
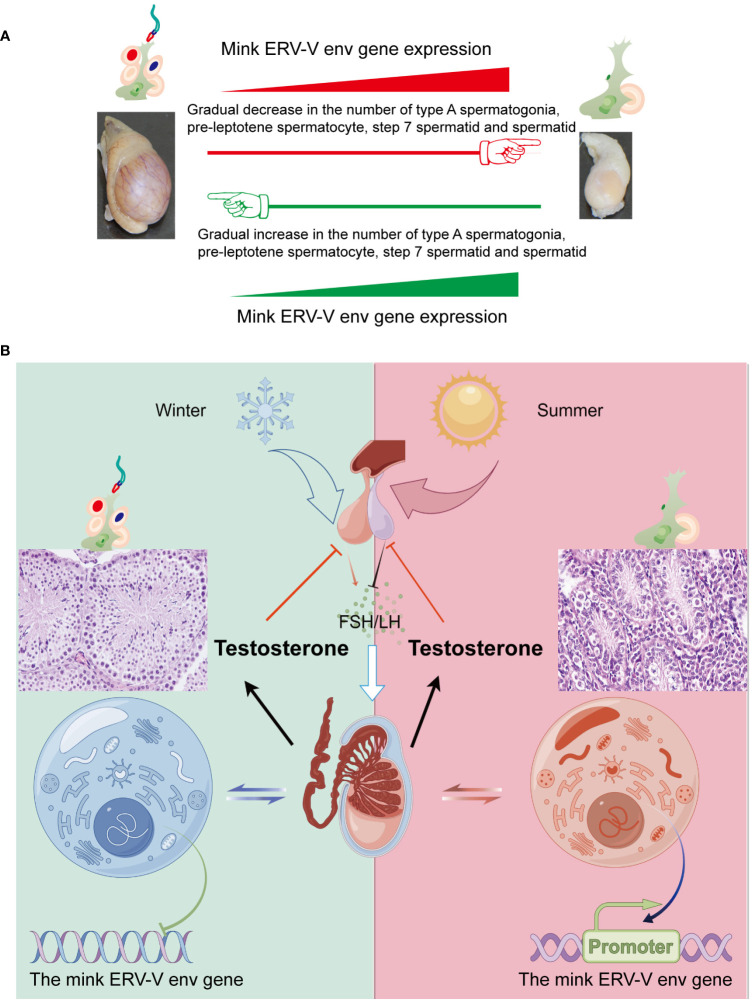
Fluctuations in the hypothalamus-pituitary-gonadal axis (HPG) lead to variations in testis size and mink ERV-env expression in minks (Neovison vison) throughout the year. **(A)** Schematic representation of the pattern of regulation of the reproductive cycle and mink ERV-env expression in minks. The expression of mink ERV-env in the testes is accompanied by cyclic changes in spermatogenesis. The expression of this gene gradually increases during the regression stage of mink testes, followed by a gradual decline during the transition to the reproductive stage. The diagram illustrates significant changes in the cellular makeup of the testes as a key factor in differences in mink ERV-V env gene expression. **(B)** Seasonal variations in the HPG axis and associated alterations in testicular activity and mink ERV-env expression are observed. Short photoperiods, which are less than 12 hours of light per day, stimulate the hypothalamus to release GnRH, leading to the production and release of FSH and LH from the pituitary gland. Gonadotrophins are responsible for supporting the production of sperms and hormones in the testes. Meanwhile, the mink ERV-V env gene switches can be fully turned off. Extended exposure to light (> 12 hours per day) suppresses the release of GnRH, ultimately halting the generation of both sperms and testosterone. In addition, the mink ERV-V env gene switches can be fully turned on. Thus, we suggest that changes in hormones can regulate the expression of the mink ERV-V env gene, causing it to transition from an active state to a less active state and then to a repressed state.

Cyclical changes in the growth and regression of testes in minks, including seasonal variations in testis size and sperm production, have been well documented (Zhang and Xu, 2022; Zhang et al., 2023). Notably, the male gonads of minks exhibit distinct characteristics during different periods, including a sexually active phase in March, followed by a period of reduced spermatogenesis and subsequent reactivation of spermatogenesis in late November (Zhang and Xu, 2022; Zhang et al., 2023). In the present study, the changes in sperm production in mature minks across seasons were strongly associated with changes in the cellular makeup of testes, as shown in **Figure 12A**. Hence, we postulated that substantial alterations in the cellular composition of testes serve as causal determinants of the differences in mink ERV-V *env* gene expression. Additionally, spermatozoan generation was significantly correlated with testosterone synthesis, and these factors displayed synchronized seasonal patterns (**Figure 12B**). It is plausible that these endocrine modifications directly affected activation and deactivation of the mink ERV-V *env* gene, resulting in fluctuations in its expression. More comprehensive investigations are required to address these questions.

We have identified a novel retroviral env gene in *Neovison vison*. Through phylogenetic and other analyses of functional motifs specific to RVs or RV subtypes, we characterized the mink ERV-V *env* gene and found it to align with class I ERV genes. Additionally, we observed a gradual increase in mink ERV-V *env* expression during the regression stage of mink testes, followed by a gradual decline during the transition to their reproductive stage. By further investigating the expression and replication of mink ERV-V *env in vitro*, we can gain further valuable insights. In particular, studies to understand the role of mink ERV-V Env in testicular regression are warranted.

## Data availability statement

The original contributions presented in the study are included in the article/**Supplementary Material**. Further inquiries can be directed to the corresponding author.

## Ethics statement

The animal study was approved by The Institutional Animal Care and Use Committee of Inner Mongolia Agricultural University, China, approved all the protocols utilized in this work (Approval number: 2020007). The study was conducted in accordance with the local legislation and institutional requirements.

## Author contributions

YZ: Conceptualization, Data curation, Formal analysis, Funding acquisition, Investigation, Methodology, Project administration, Resources, Software, Supervision, Validation, Visualization, Writing – original draft, Writing – review & editing. GW: Conceptualization, Data curation, Formal analysis, Methodology, Resources, Writing – review & editing. YaZ: Conceptualization, Data curation, Formal analysis, Funding acquisition, Investigation, Methodology, Project administration, Resources, Software, Supervision, Validation, Visualization, Writing – review & editing. XC: Data curation, Investigation, Methodology, Software, Validation, Writing – review & editing. FL: Conceptualization, Data curation, Formal analysis, Investigation, Project administration, Resources, Supervision, Writing – review & editing. HL: Formal analysis, Investigation, Methodology, Resources, Writing – review & editing. SL: Conceptualization, Data curation, Formal analysis, Funding acquisition, Investigation, Methodology, Project administration, Resources, Software, Supervision, Validation, Visualization, Writing – review & editing.

## Supplementary material

Supplementary Figure 1 Hematoxylin and eosin-stained histological sections of the annual seasonal reproductive cycle in the normal adult mink. There are three main phases of testicular function in the circannual cycle of mink: (i) testis recrudescence begins in November; (ii) Mating is observed from late February to late March; and (iii) testicular regression and a long period of sexual rest (May to October).
