# Supplementary figures and images for: Exploring the role of endogenous retroviruses in seasonal reproductive cycles: a case study of the ERV-V envelope gene in mink
Source: Front Cell Infect Microbiol. 2024 Jun 28;14:1404431. doi: 10.3389/fcimb.2024.1404431 (PMC11287128; doi:10.3389/fcimb.2024.1404431)

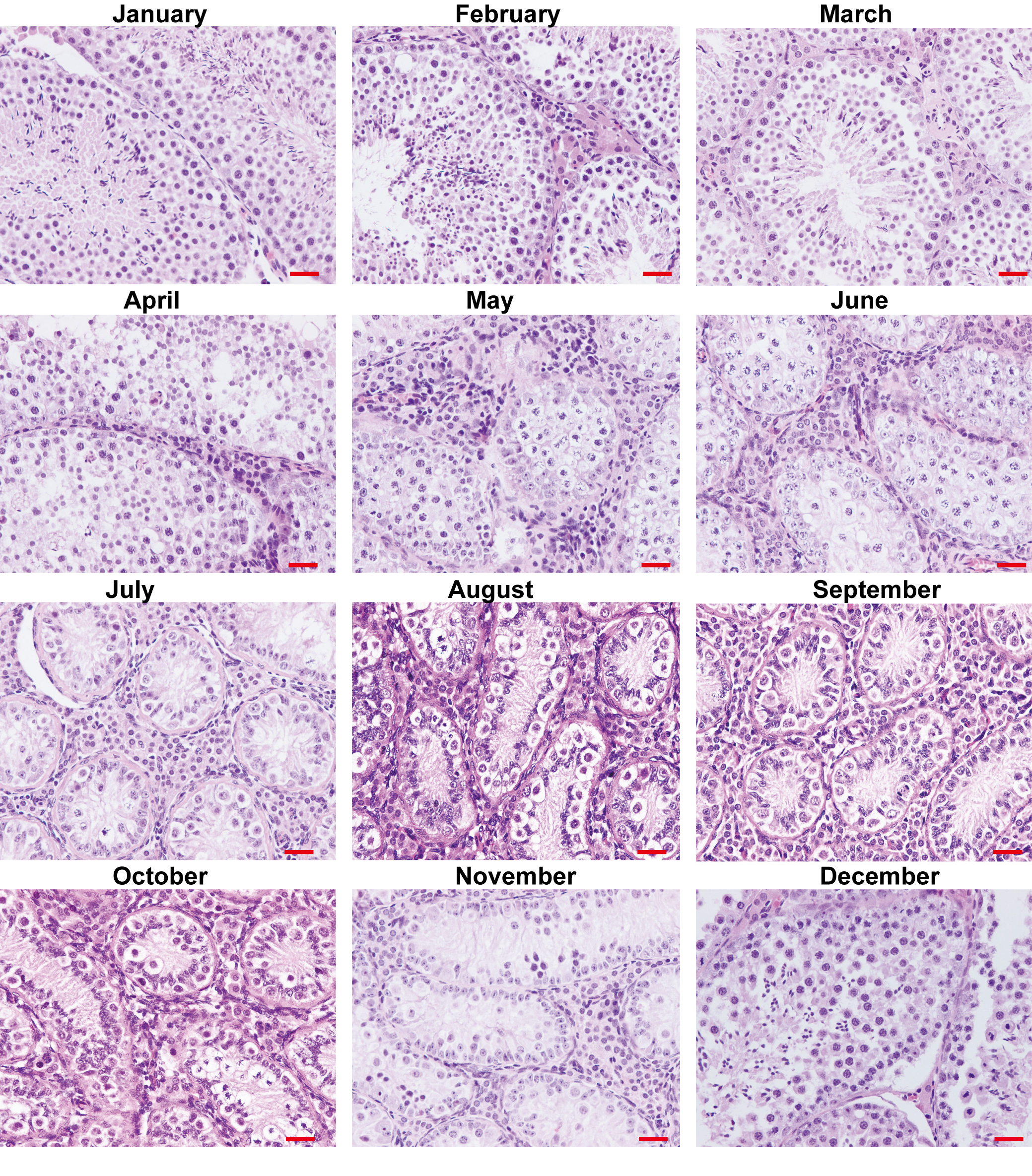

Supplement: Supplementary Figure 1 — Hematoxylin and eosin-stained histological sections of the annual seasonal reproductive cycle in the normal adult mink. There are three main phases of testicular function in the circannual cycle of mink: (i) testis recrudescence begins in November; (ii) Mating is observed from late February to late March; and (iii) testicular regression and a long period of sexual rest (May to October). [file Image_1.jpeg]
