# Supplementary material for: Exploring the role of endogenous retroviruses in seasonal reproductive cycles: a case study of the ERV-V envelope gene in mink
Source: Front Cell Infect Microbiol. 2024 Jun 28;14:1404431. doi: 10.3389/fcimb.2024.1404431 (PMC11287128; doi:10.3389/fcimb.2024.1404431)
Supplement: Supplementary file 2 [file Table_1.doc]

Table S1. Genome assemblies used in this study

| Species | Assembly Genbank Accession |
| --- | --- |
| Acinonyx jubatus (cheetah) | GCF_003709585.1 |
| Callorhinus ursinus (northern fur seal) | GCF_003265705.1 |
| Canis lupus familiaris (domestic dog) | GCF_014441545.1 |
| Caracal caracal (caracal) | GCA_016801355.1 |
| Crocuta crocuta (spotted hyena) | GCA_008692635.1 |
| Cryptoprocta ferox (fossa) | GCA_004023885.1 |
| Enhydra lutris (sea otter) | GCF_002288905.1 |
| Eumetopias jubatus (Steller sea lion) | GCF_004028035.1 |
| Felis catus (Domestic cat) | GCF_018350175.1 |
| Gulo gulo (wolverine) | GCA_900006375.2 |
| Halichoerus grypus (gray seal) | GCF_012393455.1 |
| Helogale parvula (dwarf mongoose) | GCA_004023845.1 |
| Hyaena hyaena (striped hyena) | GCF_003009895.1 |
| Lontra canadensis (Northern American river otter) | GCF_010015895.1 |
| Lutra lutra (Eurasian river otter) | GCA_902655055.2 |
| Lycaon pictus (African hunting dog) | GCA_004216515.1 |
| Lynx canadensis (Canada lynx) | GCF_007474595.2 |
| Lynx pardinus (Spanish lynx) | GCA_900661375.1 |
| Martes zibellina (sable) | GCA_012583365.1 |
| Mellivora capensis (ratel) | GCA_004024625.1 |
| Mirounga leonine (Southern elephant seal) | GCF_011800145.1 |
| Mungos mungo (banded mongoose) | GCA_004023785.1 |
| Mustela erminea (ermine) | GCF_009829155.1 |
| Mustela putorius furo (domestic ferret) | GCF_011764305.1 |
| Neogale vison (American mink) | GCF_020171115.1 |
| Neomonachus schauinslandi (Hawaiian monk seal) | GCF_002201575.2 |
| Nyctereutes procyonoides (raccoon dog) | GCA_905146905.1 |
| Odobenus rosmarus divergens (Pacific walrus) | GCF_000321225.1 |
| Otocyon megalotis (bat-eared fox) | GCA_017311455.1 |
| Panthera pardus (leopard) | GCF_001857705.1 |
| Panthera tigris (tiger) | GCF_018350195.1 |
| Paradoxurus hermaphroditus (Asian palm civet) | GCA_004024585.1 |
| Phoca vitulina (harbor seal) | GCF_004348235.1 |
| Potos flavus (kinkajou) | GCA_015708855.1 |
| Prionailurus bengalensis (leopard cat) | GCF_016509475.1 |
| Prionailurus viverrinus (fishing cat) | GCA_018119265.1 |
| Procyon lotor (raccoon) | GCA_015708975.1 |
| Proteles cristatus (Aardwolf) | GCA_017311185.1 |
| Pteronura brasiliensis (giant otter) | GCA_004024605.1 |
| Puma concolor (puma) | GCF_003327715.1 |
| Puma yagouaroundi (jaguarundi) | GCF_014898765.1 |
| Suricata suricatta (meerkat) | GCF_006229205.1 |
| Ursus americanus (American black bear) | GCA_003344425.1 |
| Ursus arctos (brown bear) | GCF_003584765.2 |
| Vulpes lagopus (Arctic fox) | GCF_018345385.1 |
| Vulpes vulpes (red fox) | GCF_003160815.1 |
| Zalophus californianus (California sea lion) | GCF_009762305.2 |
