# Supplementary material for: Exploring the role of endogenous retroviruses in seasonal reproductive cycles: a case study of the ERV-V envelope gene in mink
Source: Front Cell Infect Microbiol. 2024 Jun 28;14:1404431. doi: 10.3389/fcimb.2024.1404431 (PMC11287128; doi:10.3389/fcimb.2024.1404431)
Supplement: Supplementary file 3 [file Table_2.doc]

Table S2. Viral genomes and co-opted viral genes used in this study

| **Virus/Gene Designation** | **Genbank or Dfam Accession Number** |
| --- | --- |
| **Genbank** | |
| Bovine foamy virus (BFV) | NC_001831 |
| Bovine leukemia virus (BLV) | NC_001414 |
| Carnivora syncytin (Syn-Car1) | NM_001305592.1 |
| Desmodus rotundus endogenous retrovirus (DrEV) | NC_027117 |
| Equine foamy virus (EFV) | NC_002201 |
| Equine infectious anemia virus (EIAV) | NC_001450 |
| Feline foamy virus (FFV) | NC_039242 |
| Feline immunodeficiency virus (FIV) | NC_001482 |
| Feline leukemia virus (FeLV) | NC_001940 |
| Gibbon ape leukemia virus (GALV) | NC_001885 |
| HERVK113 | AY037928.1 |
| Human EnvV2 | NM_001191055.2 |
| Human immunodeficiency virus 1 (HIV-1) | NC_001802 |
| Human syncytin 1 (Syn1) | NM_001130925.2 |
| Human syncytin 2 (Syn2) | NM_207582.3 |
| Human T-cell leukemia virus type I (HTLV-1) | NC_001436 |
| Human T-lymphotropic virus 4 (HTLV-4) | NC_011800 |
| Jaagsiekte sheep retrovirus (JSRV) | NC_001494 |
| Koala retrovirus (KORV) | NC_039228 |
| Mouse mammary tumor virus (MMTV) | NC_001503 |
| Mouse syncytin A (SynA) | NM_001013751.2 |
| Mouse syncytin B (SynB) | NM_173420.3 |
| Murine leukemia virus (AKV) | J01998 |
| Porcine endogenous retrovirus C (PERV-C) | KY352351 |
| Rous sarcoma virus (RSV) | J02342 |
| Ruminant syncytin (Syn-Rum1) | NM_001305454.1 |
| Simian foamy virus (SFV) | NC_001364 |
| Simian immunodeficiency virus from African Green Monkey (SIVagm) | NC_001549 |
| Simian immunodeficiency virus from chimpanzee (SIVcpz) | FR686510.1 |
| Simian retrovirus 4 (SRV4) | NC_014474.1 |
| Simian T-cell lymphotropic virus 6 (STLV-6) | NC_011546 |
| Simian T-lymphotropic virus 2 (STLV-2) | NC_001815 |
| Squirrel monkey retrovirus (SMRV) | NC_001514 |
| Squirrel/marmot syncytin (Syn-Mar1) | NM_001305587.1 |
| Tenrec syncytin (Syn-Ten1) | NM_001305586.1 |
| Turkey Lymphoproliferative disease virus (MgLDV) | U09568.1 |
| Visna Maedi Virus | NC_001452 |
| Walleye dermal sarcoma virus (WDSV) | NC_001867 |
| Walleye epidermal hyperplasia virus type 1 (WEHV1) | NC_043194 |
| Walleye epidermal hyperplasia virus type 2 (WEHV2) | NC_043195 |
